# Gram-negative antimicrobial-resistant bacteria in wild and captive birds: a narrative review of ecological niches and One Health implications

**DOI:** 10.1007/s10482-026-02455-y

**Published:** 2026-11-11

**Authors:** Carolina Aparecida Ramos, Joseane Cristina Ferreira, Stella Cabral, Anelise Stella Ballaben, Rafael Antonio Casarin Penha-Filho, Ana Lúcia da Costa Darini

**Affiliations:** 1 Faculty of Pharmaceutical Sciences of Ribeirão Preto, University of São Paulo (USP), Ribeirão Preto, Brazil; 2 School of Agricultural and Veterinary Sciences, São Paulo State University (UNESP), Jaboticabal, Brazil; 3 Safe – Saúde Animal- Jaboticabal, São Paulo, Brasil

**Keywords:** Antimicrobial resistance, Gram-negative bacteria, Wild birds, Captive birds, ESBL, One Health

## Abstract

Antimicrobial resistance (AMR) in birds is increasingly investigated because wild and captive birds can encounter resistant bacteria at interfaces involving human activity, other animals, and environmental contamination. This narrative review synthesizes reports of antimicrobial-resistant Gram-negative bacteria in wild, captive, and other non-poultry avian populations. Rather than treating all detections as equivalent, the evidence is interpreted across distinct levels: bacterial occurrence or carriage, phenotypic resistance, antimicrobial resistance genes (ARGs), mobile genetic elements (MGEs), bacterial lineage relatedness, and epidemiological or experimentally demonstrated transmission. The literature shows recurrent detection of ESBL-producing *Enterobacterales*, *bla*_CTX-M_ genes, multidrug-resistant phenotypes, and diverse ARGs across multiple ecological settings. However, substantial heterogeneity in sampling, bacterial recovery, antimicrobial susceptibility testing, and molecular characterization limits direct comparison among studies and restricts inference regarding persistence, dissemination, and transmission. The central contribution of this review is therefore an ecological and molecular evidence framework that distinguishes exposure and carriage from persistence, genetic mobility, genomic relatedness, and transmission. The review also identifies methodological priorities, including harmonized sampling and susceptibility testing, longitudinal studies, cross-compartment sampling, genomic characterization, and explicit integration of ecological and epidemiological data.

**Supplementary Information:**

The online version contains supplementary material available at https://doi.org/10.1007/s10482-026-02455-y.

## Introduction

Antimicrobial resistance (AMR) is a global One Health challenge that affects humans, domestic and wild animals, and ecosystems. The emergence and spread of antimicrobial-resistant bacteria compromise the treatment of infectious diseases and are influenced by antimicrobial use, environmental contamination, microbial exchange, and interactions among different host and ecological compartments (Biswas et al. 2022). Consequently, AMR surveillance extends beyond clinical settings to animal populations and environmental compartments where resistant bacteria and resistance determinants may occur (Meier et al. 2023).

Birds are recognized as relevant to the ecology of AMR because they occupy diverse habitats and frequently interact with environments influenced by human activity. Their intestinal and cloacal microbiota can vary according to species, diet, habitat, and contact with humans and other animals (Sun et al. 2022). Free-living birds may encounter antimicrobial-resistant bacteria through contaminated water, food, soil, wastewater, agricultural environments, livestock-associated areas, and urban waste, particularly in environments affected by anthropogenic activities such as urbanization, industrialization, agriculture, and animal production (Domínguez et al. 2021). Captive birds, including those in zoos and rehabilitation centers, may acquire antimicrobial-resistant bacteria from contaminated food and water, during enclosure cleaning or veterinary procedures, or through contact with visitors, particularly in zoos (Riaz et al. 2023). These differences in ecological context may influence the occurrence of antimicrobial-resistant bacteria associated with avian populations.

Among antimicrobial-resistant microorganisms, Gram-negative bacteria deserve particular attention because of their genetic plasticity and their capacity to acquire and disseminate AMR determinants, including through horizontal gene transfer. Several clinically important taxa, including *Escherichia coli*, *Klebsiella pneumoniae*, *Salmonella* spp., *Pseudomonas* spp., *Acinetobacter* spp., and other *Enterobacterales*, are associated with multidrug-resistant infections in human and veterinary medicine and may carry clinically relevant resistance determinants (Christaki et al. 2020; World Health Organization 2024). These determinants may be chromosomal or associated with mobile genetic elements (MGEs), including plasmids and other mobile DNA structures, which can contribute to their dissemination under appropriate biological conditions (Schweizer et al. 2019).

This narrative review synthesizes relevant literature on antimicrobial-resistant Gram-negative bacteria associated with wild and captive birds, with emphasis on microbiological, ecological, epidemiological, and genomic aspects. The synthesis is interpretive rather than quantitative because the available studies differ substantially in host populations, ecological settings, sampling strategies, bacterial targets, antimicrobial susceptibility testing methods, and molecular approaches. Individual case reports and small observational studies are therefore considered primarily as evidence of occurrence rather than as population-level prevalence estimates, whereas larger observational studies are used to describe occurrence or proportions when appropriate denominators are available. Phenotypic resistance, ARG detection, MGE association, bacterial lineage relatedness, and epidemiological linkage are treated as distinct but complementary forms of evidence. Accordingly, detection of a resistant bacterium or resistance determinant is not considered, by itself, evidence of acquisition from a particular source, persistence within a bird population, horizontal transfer, or transmission between hosts or environments. Quantitative findings are reported using the original denominators whenever available, with differences in sampling design considered when comparing studies. A detailed summary of the studies included in this review, including avian setting, sample characteristics, bacterial species, phenotypic resistance, resistance determinants, MGEs, clonal information, genomic approaches, and reported relationships with human or livestock-associated isolates, is provided in Supplementary Table S1.

Despite the increasing number of studies addressing AMR in birds, important gaps remain in the integration of ecological and microbiological evidence. A broader cross-order perspective encompassing both wild and captive birds remains limited, particularly when phenotypic resistance is considered alongside ARGs, MGEs, and bacterial lineages. Recent systematic reviews have synthesized important subsets of this literature, including antimicrobial-resistant *E. coli* in wild birds globally and *Enterobacteriaceae* in European wild birds (Cocoș et al. 2025; Mbuthia and Hoza 2025). A recent review focused specifically on migratory birds and critical ARGs further highlighted flyway connectivity, anthropogenic exposure, and MGEs (Munir et al. 2027). The present review therefore does not seek to reproduce these prevalence-focused syntheses. Instead, it broadens the scope to Gram-negative bacteria beyond *E. coli*, includes captive and rehabilitation settings, and integrates ecological context with phenotypic, molecular, and genomic evidence, while distinguishing bacterial occurrence or carriage, resistance determinants, MGE association, and epidemiological links.

**Fig. 1:**
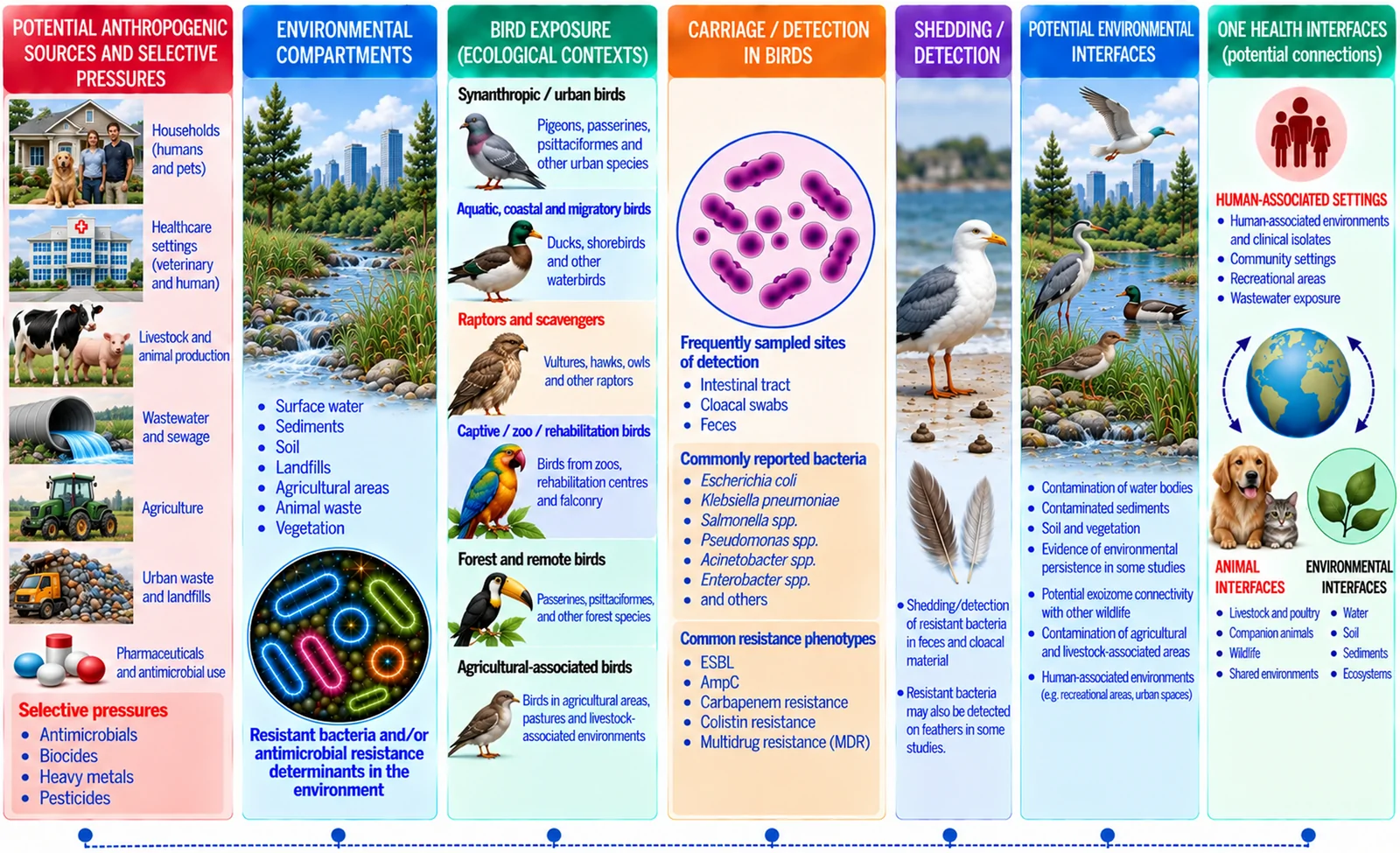
Conceptual overview of potential anthropogenic sources, environmental compartments, bird exposure contexts, detection and shedding of antimicrobial-resistant bacteria, and potential One Health interfaces. The figure illustrates possible ecological and host-associated connections

The review is organized according to ecological exposure factors rather than mutually exclusive bird categories. Factors such as urbanization, migration, feeding ecology, human management, and contact with livestock, waste, wastewater, or other contaminated environments may occur simultaneously within the same bird population and are therefore considered interconnected. This framework is used to interpret how ecological context may relate to the occurrence and distribution of antimicrobial-resistant Gram-negative bacteria across different avian groups (Fig. 1).

## Ecological niches and antimicrobial resistance in birds

Both wild and captive birds have been reported to carry antimicrobial-resistant bacteria, with the strength of the available evidence varying according to ecological context, study design, host population, and type of evidence. Detection of antimicrobial-resistant bacteria demonstrates occurrence or carriage at the time and site of sampling, whereas broader interpretations such as environmental dissemination, bacterial maintenance, or transmission require additional epidemiological or genomic evidence.

Differences among avian orders in foraging strategies, habitat preferences, and mobility, influence their exposure to and carriage of antimicrobial-resistant microorganisms. Migratory species may encounter antimicrobial-resistant bacteria across different environments during their movements, and their mobility provides an ecological context in which resistant bacteria may be detected across geographically distant areas. (Lorenti et al. 2021). Ground-foraging and scavenging birds are also noteworthy, as they often inhabit agricultural zones and livestock facilities, where they may interact directly or indirectly with poultry, pigs, and ruminants. Such environments provide opportunities for exposure to antimicrobial-resistant bacteria and represent ecological interfaces in which resistant bacteria and associated resistance determinants may occur. From a One Health perspective, birds can function as ecological sentinels of environmental AMR and may encounter resistant bacteria across interconnected habitats through their movements and ecological interactions. These characteristics make avian populations relevant to AMR surveillance, particularly at interfaces between wildlife, human-impacted environments, and other animal populations. However, detection of resistant bacteria in birds should not, in the absence of additional epidemiological or genomic evidence, be interpreted as evidence that birds transmit these bacteria between hosts or environments (Khan et al. 2020).

A recent global systematic review and meta-analysis of antimicrobial-resistant *Salmonella* in wild birds highlights the broad geographic and ecological variation in resistance patterns reported across avian populations (Ntakiyisumba et al. 2026). These findings emphasize the importance of considering ecological context when interpreting AMR in wild birds. Accordingly, comparisons across urban, aquatic, agricultural, migratory, and lower-anthropogenic-influence settings may help characterize differences in resistance patterns associated with distinct exposure contexts.

### Urban and synanthropic birds

Synanthropic birds frequently forage in urban and agricultural environments where food waste, wastewater, grain storage, refuse, and human-associated surfaces may provide opportunities for exposure to resistant bacteria. Passerines, pigeons, and some psittacines have therefore been investigated as indicators of AMR at human–wildlife interfaces (Martín-Maldonado et al. 2022).

Passeriformes are frequently encountered in human-modified environments and are also represented among birds involved in the illegal wildlife trade. In this review, multidrug resistance (MDR) refers to resistance to at least one antimicrobial agent in three or more antimicrobial classes (Magiorakos et al., 2012). Studies involving trafficked Passeriformes have reported MDR *E. coli* isolates (Matias et al. 2016; Silva et al. 2021). Additionally, *K. pneumoniae* isolated from healthy Passeriformes confiscated during illegal wildlife trade in Brazil carried genes associated with quinolone and fluoroquinolone resistance (Davies et al. 2016). *Klebsiella* spp., *Enterobacter* spp., *Pantoea agglomerans*, *Serratia* spp., and *Proteus mirabilis*, all exhibiting MDR profiles, have been identified in cloacal swabs and necropsies of canaries (*Serinus canaria*) from various properties in Fortaleza, Brazil (Horn et al. 2015). A study conducted in the Caatinga biome (Ceará, Brazil) analyzed cloacal swabs from 50 wild birds, including the Ceara Gnatheter (*Conopophaga cearae*) and the Red-necked Tanager (*Tangara cyanocephala*), which are classified as vulnerable (VU) by the Brazilian Ministry of the Environment. That study identified 55 *Enterobacteriaceae* isolates, mainly *Pantoea agglomerans* (36%) and *E. coli* (26%). Resistance was most frequently detected to ampicillin (41.8%) and nalidixic acid (36.3%), while 23.5% of isolates were MDR (Beleza et al. 2024).

A recent study in the United Kingdom found MDR bacteria in 75% of fecal samples. *E. coli* was detected in all birds, while *Campylobacter* spp. and *Salmonella* spp. were detected in 18.9% and 11.6% of birds, respectively. MDR rates were 59% for E. coli, 88% for *Campylobacter* spp, and 70% for *Salmonella* spp. Younger birds and species living in proximity to humans, such as house sparrows (*Passer domesticus*) and blackbirds (*Turdus merula*), were more frequently associated with MDR bacterial carriage. Widespread shedding of MDR bacteria by passerines may contribute to environmental contamination and represents a potential interface for zoonotic exposure (Dunn and Clegg 2025).

Psittacines are frequently kept in captivity and are also represented among birds involved in the illegal wildlife trade. In Brazil, *E. coli* strains resistant to ciprofloxacin, ertapenem, and ceftazidime have been isolated from rescued psittacines associated with the illegal wild bird trade. The isolates carried the *bla*_KPC_ gene, a carbapenemase-encoding resistance determinant, and exhibited resistance to carbapenems (Matias et al. 2016; Silva et al. 2021).

Columbiformes, particularly domestic and urban pigeons (*Columba livia*), have also been investigated for antimicrobial-resistant bacteria. MDR *E. coli* was isolated from domestic pigeons found near grain-storage silos. In the same study, *Salmonella* Typhimurium was isolated but remained susceptible to the tested antimicrobials despite carrying ARGs, illustrating that the detection of resistance determinants does not necessarily correspond to a resistant phenotype (Carvalho et al. 2020). Additionally, MDR *Hafnia alvei* and *Pantoea ananatis* were isolated from fecal samples of urban pigeons (*Columba livia* f. urbana) in France (Ngaiganam et al. 2019). In 2021, a study conducted in Qingdao, China, analyzed 149 fecal samples from homing pigeons, ornamental pigeons, and meat pigeons, resulting in 149 *E. coli* isolates. Among these, 24.8% exhibited an MDR profile. Additionally, two isolates were identified as carriers of Extended-Spectrum Beta-Lactamase (ESBL) genes (Wang and Hu 2022).

European studies have also documented AMR among diverse wild bird populations. *E. coli* isolates from several common European wild bird species, including synanthropic birds, birds of prey, waterfowl, and passerines, were investigated and multidrug-resistant phenotypes involving several antimicrobial classes were identified. The study also investigated selected resistance-associated genes, providing evidence of antimicrobial-resistant *E. coli* across distinct avian ecological groups (Guenther et al. 2010).

Taken together, studies involving synanthropic and small-bodied birds show recurrent detection of antimicrobial-resistant Gram-negative bacteria in human-impacted environments. MDR and ESBL-associated phenotypes have been reported in passerines, psittacines, and pigeons, with resistance determinants including *bla*_CTX-M_ and *bla*_KPC_ genes identified in some isolates. However, the available studies differ substantially in host populations, sampling strategies, bacterial targets, and antimicrobial susceptibility-testing methods, limiting direct comparison of resistance frequencies across studies. The repeated detection of resistant bacteria in urban and other human-associated settings supports the relevance of these birds for AMR surveillance.

### Aquatic, coastal and migratory birds

Aquatic and migratory birds use wetlands, rivers, estuaries, coastal habitats, landfills, and other environments that can receive anthropogenic contaminants. These habitats may expose birds to diverse bacterial communities originating from environmental, human-associated, and animal-associated sources. Migratory movements may further connect geographically separated habitats and populations, making aquatic and migratory birds relevant for investigating the occurrence and geographic distribution of antimicrobial-resistant bacteria.

Migratory birds in South Korea were found to carry *Salmonella enterica* serovars, including strains resistant to ciprofloxacin and genetically related to poultry and meat isolates by pulsed-field gel electrophoresis (PFGE) typing. These findings indicate genetic relatedness between isolates from migratory birds and those recovered from poultry and meat, providing evidence of an ecological interface between these hosts and food-associated environments (Wei et al. 2020). In Brazil, migratory white-faced whistling-duck (*Dendrocygna viduata*) carried SPM-1-producing *P. aeruginosa* ST277, indicating that clinically important carbapenemase-producing bacteria can occur in apparently healthy wild birds (Martins et al. 2018).

A recent study in Pakistan detected multidrug-resistant *Pseudomonas aeruginosa* in captive birds, including ducks, documenting the occurrence of resistant bacteria within zoo environments and its relevance to One Health surveillance (Nisar Khan et al. 2025).

A study of migratory birds in China identified *E. coli* carrying *tet*(X4), *bla*_NDM_, and *mcr-1*, with several resistance plasmids showing conjugative potential (Zhang et al. 2022). A recent global review similarly identified *bla*_NDM_, together with *bla*_CTX-M_, *bla*_KPC_, *mcr-1*, and *tet*(X4), among clinically important resistance determinants reported in migratory birds, while emphasizing substantial variation in the strength of evidence across studies (Munir et al. 2027).

Gulls are particularly well studied because they frequently occupy human-impacted environments. Studies have reported the isolation of predominantlytetracycline-resistant *Proteus mirabilis* from gulls, together with *Enterobacterales* resistant mainly to third-generation cephalosporins, and carrying ARGs, including *bla*_CTX–M–14_, *bla*_CTX-M–15_, *bla*_CTX–M–55_, *bla*_CMY–2_ and *bla*_OXA-48_ (Zeballos-Gross et al. 2021).

Comparative studies have shown that resistance patterns can differ between avian populations. Studies identified differences in ESBL prevalence and CTX-M genotype profiles between Franklin’s gulls (*Leucophaeus pipixcan*) sampled in Canada and Chile, suggesting that local anthropogenic exposure contributes to the observed patterns (Bonnedahl et al. 2015). More recent genomic evidence indicates that migratory movements may also contribute to the geographic redistribution of clinically relevant antimicrobial-resistant bacteria.

Coastal monitoring in Brazil provides further evidence that migratory status alone may not explain AMR patterns in seabirds. *E. coli* was isolated in the majority of sampled seabirds, with resistance to at least one antimicrobial detected in many isolates and MDR profiles in a subset. Importantly, no significant differences in AMR profiles were observed between migratory and resident birds, suggesting that local ecological and environmental factors may contribute substantially to resistance patterns in coastal bird populations. (Cardoso et al. 2023).

A *Salmonella enterica* serovar Agona isolate was recovered from the cloaca of a silver gull (*Chroicocephalus novaehollandiae*) in Australia. Phenotypically, the isolate showed resistance to 12 antibiotics spanning nine classes and was classified as extensively drug-resistant (XDR) (Cummins et al. 2020). In South Asia, wild birds sampled atthe Trimmu Barrage in Pakistan carried ESBL-producing *E. coli*, *Enterobacter cloacae*, and *Klebsiella pneumoniae*, with *bla*_CTX-M_ and *bla*_TEM_ among the detected resistance determinants (Saeed et al. 2023).

Studies from Africa also provide evidence of AMR among wild and migratory birds. In Egypt, colistin resistance genes *mcr-1* and *mcr-2* were detected in Enterobacteriaceae isolated from resident and migratory wild birds. The same study also detected these genes in surface water and human samples collected from associated environments, illustrating the occurrence of clinically relevant resistance determinants across wildlife, environmental, and human-associated compartments (Ahmed et al. 2019).

Taken together, studies of aquatic, coastal, and migratory birds indicate that antimicrobial-resistant Gram-negative bacteria occur across geographically and ecologically distinct populations. However, the evidence does not support a uniform role for migration in AMR dissemination. Differences between migratory and resident populations suggest that local environmental exposure can influence resistance patterns, whereas genomic studies provide evidence that some birds may carry resistant lineages across geographically separated habitats. Thus, migration should be considered a potential mechanism of geographic connectivity rather than direct evidence of transmission, with the epidemiological significance of carriage depending on the resistance phenotype, genetic determinants, bacterial lineage, and evidence of source or transmission linkage.

### Raptors and scavengers

Raptors and scavengers may encounter antimicrobial-resistant Gram-negative bacteria through predation, consumption of animal carcasses, and contact with human-impacted environments. Their feeding behaviour can provide opportunities for exposure to bacteria originating from livestock, waste, and other anthropogenic sources. However, the presence of resistant bacteria in these birds does not, by itself, demonstrate acquisition from a specific source or subsequent transmission.

Recent studies in free-living raptors have documented the carriage of clinically relevant antimicrobial-resistant bacteria. In southwestern Siberia, 51 *E. coli* isolates were recovered from 35 of 55 free-living black kites (*Milvus migrans*), most of which exhibited multidrug-resistant profiles. Among the whole-genome-sequenced isolates, 75% of those showing ESBL- or AmpC-associated resistance and carrying the *mcr-1* gene also harbored IncI2 plasmids. These isolates were detected in birds inhabiting areas close to large cities. These findings demonstrate that free-living raptors can carry clinically important resistant *E. coli* and suggest that proximity to human-influenced environments may contribute to their exposure (Tarabai et al. 2023).

Scavenging behaviour represents another potential exposure pathway, particularly in settings where vultures consume livestock carcasses. Among French griffon vultures (*Gyps fulvus*) feeding on carcasses from extensive livestock farms, 11.8% of recovered bacteria were resistant to extended-spectrum cephalosporins, including five ESBL-producing and one AmpC-producing *E. coli*. The ESBL-producing isolates were clonal and carried *bla*_CTX-M-15_ (Haenni et al. 2023).

Similar findings have also been reported in African vultures. In hooded vultures (*Necrosyrtes monachus*) sampled in the Gambia, 54 ESBL-producing *E. coli* isolates and five ESBL-producing *Klebsiella pneumoniae* isolates were recovered from 100 fecal samples. Among the 52 sequenced isolates, *bla*_CTX-M_ genes predominated, with *bla*_CTX-M-15_ detected in 46 isolates (Woksepp et al. 2023).

Landfills may represent another important exposure setting for scavenging birds. In northeastern Spain, Eurasian griffon vultures (*Gyps fulvus*) foraging at a landfill carried extended-spectrum cephalosporin-resistant *E. coli*, with a high proportion of isolates showing MDR. The predominance of *bla*_CTX-M-15_ and the occurrence of high-risk *E. coli* lineages previously reported in humans suggest that landfills may facilitate contact between vultures and clinically relevant resistant bacteria (Guitart-Matas et al. 2024).

Recent investigations of New World vultures further demonstrate that AMR varies according to ecological context. Sampling conducted at roosts, landfills, and boneyards in 2023–2024 recovered *E. coli* and *Salmonella* spp. from vulture-associated environments, with resistance to aminoglycosides, cephalosporins, and tetracyclines. MDR was detected in 5.3% of *E. coli* and 17.6% of *Salmonella* spp. isolates, and resistance profiles varied according to sampling site, season, and sample type. Notably, MDR *E. coli* and *Salmonella* spp. were particularly associated with landfill and boneyard samples, suggesting that waste-associated environments may represent important exposure points for scavenging birds (Tallon et al. 2024). However, the study did not demonstrate direct transmission of resistant bacteria between waste sites and vultures.

Overall, recent evidence indicates that free-living raptors and scavengers can carry clinically relevant antimicrobial-resistant bacteria, particularly ESBL-producing bacteria. Across studies, livestock carcasses, landfills, boneyards, and other human-influenced environments emerge as recurring contexts of exposure. These observations support the ecological relevance of raptors and scavengers in AMR surveillance.

### Captive, zoo, and rehabilitation birds

Captive, zoo, and rehabilitation settings differ from free-ranging environments because birds may experience changes in diet, housing density, antimicrobial exposure, and contact with humans, other animals, and shared environments. In this review, captive birds are defined as wild or domesticated birds maintained under human care for prolonged or permanent periods, including birds kept in zoos, aviaries, conservation facilities, and falconry, whereas rehabilitation birds refer to free-living wild birds temporarily admitted to wildlife rescue or rehabilitation centers for medical treatment, recovery, or subsequent release. These conditions may influence the occurrence and persistence of antimicrobial-resistant Gram-negative bacteria and provide opportunities for their maintenance or exchange within managed environments.

Captive and rehabilitation centers may influence the AMR profiles of birds during their stay. Recent evidence from Brazil illustrates the occurrence of resistant Gram-negative bacteria among both captive and rescued wild birds maintained in a zoological setting. Among 80 healthy birds investigated, cefotaxime-resistant Gram-negative bacilli were recovered from 26 zoo birds and 18 rescued wild birds. *E. coli* and *Klebsiella* spp. were the predominant bacteria isolated. Among the 65 isolates recovered, 75% were multidrug-resistant, and most were classified as ESBL producers. The *bla*_CTX-M-group-1_, *bla*_TEM_, and *bla*_SHV_ were the most frequently detected resistance genes, while *bla*_KPC_ was identified in a *Klebsiella pneumoniae* isolate. PFGE and plasmid analysis also revealed genetic similarities and the presence of plasmids among isolates from birds without known direct contact, suggesting that resistant Gram-negative bacteria may persist and circulate within managed wildlife environments (Ramos et al. 2024).

*E. coli* was isolated from 121 raptors and 51 synanthropic wild birds admitted to a wildlife rescue center in Italy, with samples collected at admission and during the first 10 days following admission. MDR *E. coli* was detected in 39.6% of the animals, while ESBL-producing isolates were recovered from 13.2%. An increase in the number of resistance phenotypes was observed during the first 10 days after admission, particularly among birds receiving antimicrobial treatment, indicating temporal changes in bacterial carriage during the rehabilitation period (Prandi et al. 2023).

Another report recovered 389 *Enterobacteriaceae* isolates from 61 raptors in a raptor rehabilitation center in the southeastern United States and evaluated susceptibility to nine antimicrobials. Resistance was most frequently detected to tetracycline and ampicillin, while 6.3% of isolates were multidrug-resistant. Resistance profiles varied according to rehabilitation time, antimicrobial exposure, diet, and health outcome, whereas birds released back into the wild showed resistance patterns similar to those observed at admission. These findings suggest that resistance profiles during rehabilitation may reflect both pre-existing bacterial carriage and changes associated with treatment and management conditions (Stahl et al. 2025).

A recent study of captive birds of prey in Portugal further demonstrated the occurrence of antimicrobial-resistant *E. coli* in managed raptors. Eighty-four *E. coli* isolates were obtained from 27 captive birds of prey used in falconry and related activities. The isolates showed resistance to several antimicrobials, and ESBL production was also detected. ERIC-PCR revealed substantial genetic diversity among the isolates, indicating that the resistant *E. coli* population was not represented by a single dominant clone. Raw food, animal management, and contact with other animals may represent potential exposure pathways, although these routes were not directly demonstrated (Magalhães et al. 2024).

Evidence from zoological gardens in other geographic regions indicates that resistant *E. coli* also occurs among captive wild birds outside Europe and South America. In a study of captive animals at Seoul Zoo, *E. coli* was recovered from 58 of 61 animals, and 62% of the tested *E. coli* isolates exhibited MDR. Multiple sequence types were identified, with several lineages previously reported in human or livestock-associated contexts (Kim et al. 2023). More recently, ESBL-producing *E. coli* were investigated in captive wild birds from zoological gardens in Nigeria. Among 50 pooled fecal samples, 26 ESBL-producing *E. coli* isolates were identified. The *bla*_CTX-M_, *bla*_TEM_, and *bla*_SHV_ genes were also detected (Adesola et al. 2025). These findings demonstrate that captive wildlife can carry clinically relevant resistant Gram-negative bacteria across geographically distinct zoological settings, although differences in sampling design and antimicrobial susceptibility methods limit direct comparisons among studies.

### Forest and remote environments

Forest and remote environments are generally characterized by lower direct anthropogenic influence than urban, agricultural, or captive settings, although their degree of isolation from human-associated sources of AMR can vary considerably. Birds inhabiting these environments may nevertheless encounter antimicrobial-resistant Gram-negative bacteria through contaminated water, soil, food resources, or occasional contact with human-impacted areas. Evidence from these settings is particularly relevant for assessing whether AMR occurs in wildlife populations with comparatively limited direct exposure to antimicrobial use.

Recent evidence from Brazil demonstrates the occurrence of antimicrobial-resistant *Enterobacterales* among free-living birds in the Caatinga biome. Fifty birds representing 28 species were investigated, and 55 *Enterobacteriaceae* isolates were recovered, with *Pantoea agglomerans* and *E. coli* being the most frequently isolated species. Resistance was most frequently detected to ampicillin (41.8%), followed by nalidixic acid (36.3%) and amoxicillin–clavulanic acid (32.7%). MDR was identified in 23.5% of the isolates, including strains resistant to up to seven antimicrobial classes. The study included species from different families associated with forest and semi-arid habitats, demonstrating that resistant *Enterobacterales* occur among free-living birds in a tropical biome outside the settings traditionally associated with intensive antimicrobial use (Beleza et al. 2024).

Genomic evidence has further demonstrated the occurrence of clinically relevant resistant *E. coli* among wild birds in South America. Two ESBL-producing *E. coli* isolates carrying *bla*_CTX-M-55_ or *bla*_CTX-M-65_ were recovered from a creamy-bellied thrush (*Turdus amaurochalinus*) in Brazil and a variable hawk (*Geranoaetus polyosoma*) in Chile. Both isolates belonged to the globally distributed ST602 lineage and carried additional resistance determinants beyond beta-lactams, including genes associated with resistance to heavy metals, disinfectants, and pesticides. Phylogenomic comparisons showed relatedness between the bird-associated isolates and *E. coli* genomes from environmental, livestock, poultry, and human-associated sources, although the direction and source of acquisition could not be established (Dalazen et al. 2023).

More recent evidence from Brazil has also identified ESBL-producing *E. coli* in tropical seabirds inhabiting protected coastal environments. In magnificent frigatebirds (*Fregata magnificens*) from the Cagarras Islands, one ceftriaxone-resistant *E. coli* isolate carried *bla*_CTX-M-15_ on an IncB/O/K/Z plasmid and belonged to ST5614. Although only one resistant isolate was recovered from 15 birds, its detection in a protected island environment illustrates that clinically relevant resistance determinants may occur even in wildlife populations with limited direct exposure to antimicrobial treatment (Pribul et al. 2025).

In a remote area of Chilean Patagonia, ESBL-producing *E. coli* carrying *bla*_CTX-M-1_ and *bla*_CTX-M-55_ were detected in gulls. The isolates belonged to internationally distributed sequence types and showed phylogenomic relatedness to *E. coli* from environmental, livestock, and companion-animal sources in the United States, indicating that clinically relevant resistance determinants can occur in birds inhabiting remote environments (Fuentes-Castillo et al. 2023).

### Agricultural environments

Agricultural environments provide frequent contact between wild birds, livestock, animal waste, feed, soil, and surface water, creating opportunities for exposure to antimicrobial-resistant Gram-negative bacteria. However, detection of resistant bacteria in birds using these environments does not by itself establish transmission between livestock and wildlife. Studies that sample multiple compartments of the same agricultural system provide stronger evidence for assessing bacterial relatedness and possible exchange between hosts.

A study conducted on 54 Swedish dairy farms investigated antimicrobial-resistant *E. coli* by sampling calves, wild birds, rodents, flies, manure drains, and manure pits. Whole-genome sequencing (WGS) identified resistance genes, plasmids, and closely related isolates among different sources, including instances involving birds and other farm-associated compartments. The study therefore provided genomic evidence of shared *E. coli* lineages within agricultural environments, although the direction of transmission could not be established. The generally low levels of AMR observed were also consistent with the limited antimicrobial use practices on the farms (Hickman et al. 2022). In a UK outdoor pig farm with low antimicrobial use, *E. coli* was detected from pigs and seagull feces over one year. WGS identified closely related isolates, including isolates from pigs and wild birds separated by fewer than 14 single-nucleotide polymorphisms, supporting epidemiological linkage within the farm environment (Storey et al. 2022).

Evidence synthesized by the European Food Safety Authority (EFSA**)** also indicates that wild birds may carry antimicrobial-resistant bacteria and clinically relevant resistance determinants at interfaces involving human and animal-associated environments. Reported examples include carbapenem-resistant *Salmonella Corvallis* carrying *bla*_NDM-1_ and third-generation cephalosporin-resistant *E. coli* and *Klebsiella* spp. carrying beta-lactamasegenes, including *bla*_CTX-M-15_ and *bla*_CMY-2_. These findings further illustrate the occurrence of clinically relevant resistance determinants at wildlife–livestock and wildlife–environment interfaces (Koutsoumanis et al. 2021).

These studies illustrate the value of agricultural settings for investigating the ecology of AMR beyond detection in individual bird populations. However, the presence of genetically related *E. coli* in birds and livestock should not be interpreted as evidence of a defined transmission route.

## Molecular ecology of antimicrobial resistance in avian-associated Gram-negative bacteria

The molecular basis of AMR in avian-associated Gram-negative bacteria involves enzymatic inactivation of antimicrobials, modification of antimicrobial targets, changes in membrane permeability, and active efflux. Among these mechanisms, beta-lactamase production and the acquisition of resistance genes through MGEs have been most extensively characterized in the studies reviewed. Molecular characterization is therefore relevant not only for identifying resistance determinants, but also for assessing their genetic context, potential mobility, and distribution among bacterial lineages.

### Beta-lactamases and carbapenemases

Beta-lactamases are among the most frequently reported resistance determinants in avian-associated Gram-negative bacteria. Extended-spectrum beta-lactamase (ESBL)-associated genes, particularly *bla*_CTX-M_, have been identified in *E. coli* and other *Enterobacterales* from birds in different ecological settings. Within the CTX-M family, *bla*_CTX-M-15_ has been repeatedly detected, while *bla*_CTX-M-1_, *bla*_CTX-M-55_, and *bla*_CTX-M-65_ have also been reported. Other beta-lactamase determinants, including *bla*_TEM_, *bla*_SHV_, *bla*_CMY_, and *bla*_OXA_ variants, occur either alone or in combination with other resistance genes. The distribution of these determinants across different bacterial species and avian environments indicates that resistance to extended-spectrum beta-lactams is not restricted to a single ecological niche (Fuentes-Castillo et al. 2019, 2023; Woksepp et al. 2023; Guitart-Matas et al. 2024; Ramos et al. 2024).

Beta-lactamase-mediated resistance has been documented across diverse avian populations and geographic regions. In Bangladesh, antimicrobial-resistant and ESBL-producing *E. coli*, including isolates carrying the *bla*_CTX-M_ gene, were reported in wild birds in a study that also included and free-ranging poultry (Hasan et al. 2012). In Japan, resistant *E. coli* carrying the *bla*_CTX-M_ gene and AmpC beta-lactamase genes were identified among several wild bird species (Asai et al. 2025). Similar resistance determinants have also been reported in wild and migratory birds from India and Nigeria (Afiukwa et al. 2016; Raghav et al. 2022). Together, these studies illustrate the broad geographic distribution of ESBL-producing and AmpC-associated resistance among avian *E. coli*.

Beta-lactamase families comprise enzymes with different hydrolytic profiles. This is particularly relevant for ESBLs, which include enzymes with variable activity against extended-spectrum beta-lactams (Palzkill 2018). Therefore, detection of a specific beta-lactamase gene indicates the presence of a resistance determinant but does not necessarily predict the phenotypic spectrum of beta-lactam resistance.

Carbapenemase-encoding genes were less frequently reported in the included reports than ESBL-associated determinants, but clinically important genes such as *bla*_KPC_ and *bla*_NDM_ were identified. The *bla*_KPC_ gene has been detected in *E. coli* and *Klebsiella pneumoniae* isolates from rescued Psittaciformes, whereas *bla*_NDM_ has been identified in *E. coli* associated with migratory birds. The occurrence of *bla*_SPM-1_ in *Pseudomonas aeruginosa* from migratory birds further illustrates that clinically important metallo-beta-lactamases may also occur outside *Enterobacterales*. These findings expand the range of resistance mechanisms documented in avian-associated Gram-negative bacteria, although their detection does not establish dissemination between birds or between avian and non-avian hosts (Martins et al. 2018; Silva et al. 2021; Zhang et al. 2022; Ramos et al. 2024).

The clinical significance of these determinants also depends on their genetic context and expression. Detection of a beta-lactamase gene does not necessarily predict the corresponding resistance phenotype, and phenotypic resistance may result from mechanisms not assessed by targeted molecular assays. Differences in gene expression, gene integrity, chromosomal mutations, permeability, efflux, and susceptibility-testing criteria may contribute to genotype–phenotype discordance. In *Salmonella* Typhimurium from domestic pigeons, for example, resistance-associated genes were detected in isolates that remained susceptible to the corresponding antimicrobials. Molecular detection should therefore be interpreted together with phenotypic susceptibility testing rather than as a direct substitute for it (Carvalho et al. 2020).

### Plasmids and horizontal gene transfer

Plasmids provide an important genetic context for AMR because they can carry multiple resistance determinants and, when conjugative or mobilizable, can mediate their horizontal transfer between bacterial cells. Plasmids belonging to IncF, IncI, IncX, IncHI2, and IncN groups have been identified among avian-associated Gram-negative bacteria (Munir et al. 2027). The presence of these replicons indicates that resistance determinants may occur within genetic elements capable of persistence and, in some cases, transfer, but plasmid detection alone does not establish horizontal transmission in natural environments.

Examples from avian-associated isolates include *bla*_NDM-4_, *tet*(X4), and *mcr-1* identified in plasmids from migratory birds, with experimental conjugation demonstrating transfer potential for the plasmid under laboratory conditions. Such experiments provide evidence that the genetic elements are capable of transfer, but this should be distinguished from demonstrated transmission between birds, humans, or environmental compartments in the field (Zhang et al. 2022).

The interpretation of plasmid-mediated resistance therefore requires consideration of both the resistance determinant and its surrounding genetic context. Plasmid replicon typing, characterization of conjugation-associated genes, and comparison of plasmid sequences can provide stronger evidence regarding the potential mobility and persistence of ARGs than detection of the resistance gene alone.

### Integrons, transposons, and insertion sequences

Resistance genes may also be embedded within more complex mobile genetic structures. Integrons can capture and express resistance gene cassettes, whereas transposons and insertion sequences can contribute to the movement of resistance regions within and between replicons. These elements may occur in nested arrangements in which a resistance gene is associated with an insertion sequence or transposon located on a plasmid, increasing the complexity of the genetic context in which AMR is maintained.

This organization has been described in avian-associated isolates. For example, *bla*_KPC-2_ in a *K. pneumoniae* isolate from a rescued free-living bird was located on an IncN15 plasmid within a *Tn*4401b-like structure. Such an arrangement provides evidence of a defined genetic context for the carbapenemase gene and is more informative than reporting *bla*_KPC-2_ alone. However, the identification of a mobile genetic structure indicates the potential for genetic mobility and does not demonstrate that the resistance region was transferred between hosts in the environment (Ramos et al. 2026).

European studies have also documented AMR and resistance-associated genetic determinants in gulls. In one study,257 *E. coli* isolates from young Black-headed Gulls (*Larus ridibundus*) in three nesting colonies in the Czech Republic were investigated. Resistance to at least one antimicrobial was detected in 29.9% of the isolates, and nine resistant isolates carried class 1 integrons (Dolejska et al. 2007).

### High-risk bacterial clones

Antimicrobial resistance may also disseminate through expansion of successful bacterial lineages rather than exclusively through horizontal transfer of individual ARGs. Sequence typing and whole-genome analysis have identified avian-associated *E. coli* belonging to sequence types that have also been reported in humans, livestock, companion animals, and environmental sources (Fuentes-Castillo et al. 2019, 2023; Massella et al. 2020; Ewbank et al. 2022; Dalazen et al. 2023).

The identification of the same sequence type in different host populations is evidence of shared lineage membership, but it should not be interpreted as direct evidence of transmission. Sequence types may be widely distributed and may persist across multiple reservoirs over extended periods. Inference requires higher-resolution genomic comparisons together with compatible spatial, temporal, and epidemiological information. This distinction is particularly important when comparing avian isolates with bacteria recovered from livestock, humans, or environmental sources.

### Co-selection and multidrug resistance

Multidrug resistance in avian-associated Gram-negative bacteria frequently reflects the accumulation of several resistance determinants within the same isolate. This may result from the acquisition of plasmids carrying multiple ARGs, the accumulation of MGEs, or chromosomal mechanisms affecting several antimicrobial classes. Consequently, selection for resistance to one antimicrobial may maintain additional resistance determinants when they occur within the same genetic background.

Co-selection may also involve selective pressures other than antimicrobials. Some avian-associated isolates have carried determinants associated with resistance or tolerance to metals, disinfectants, or pesticides in addition to ARGs. In ESBL-producing *E. coli* from wild birds, for example, resistance determinants associated with heavy metals, disinfectants, and pesticides were detected alongside ARGs. Such findings indicate that environmental selective pressures may contribute to the maintenance of multidrug-resistant bacteria, although the contribution of each selective factor cannot be inferred from gene detection alone (Dalazen et al. 2023).

### Resistome versus cultured-isolate approaches

The molecular profile obtained from avian-associated bacteria depends on the methodological approach used. Culture-based studies allow resistance phenotypes to be linked to viable bacterial isolates and enable subsequent characterization of sequence types, plasmids, resistance genes, and other genomic features. However, they are restricted to bacteria that can be recovered under the culture conditions used and may therefore underestimate the diversity of resistance determinants present in the sampled community.

Metagenomic approaches provide a broader view of the resistome by allowing the detection of ARGs from culturable and non-culturable organisms. Metagenomic analysis of Himalayan vultures, for example, identified a diverse collection of ARGs and associations between resistance determinants and MGEs. However, detection of an ARG by metagenomics does not necessarily identify its bacterial host, demonstrate phenotypic expression, or establish that horizontal transfer has occurred. Culture-based and metagenomic approaches therefore provide complementary rather than interchangeable information (Zhai et al. 2024).

This distinction is relevant when comparing studies conducted in different ecological settings. The higher number or diversity of ARGs detected by metagenomics cannot be directly compared with the prevalence of resistant isolates recovered by culture without accounting differences in the biological material analyzed, sequencing depth, analytical pipelines, and resistance definitions.

### Whole-genome sequencing and genomic epidemiology

WGS provides the highest genomic resolution among the approaches used in the reviewed studies and can integrate information on ARGs, sequence types, chromosomal mutations, plasmid replicons, transposons, insertion sequences, and other accessory-genome components. These data allow resistance determinants to be examined together with the bacterial genetic background and can help distinguish, at least in part, between dissemination of resistance genes and dissemination of bacterial lineages (Hickman et al. 2022; Storey et al. 2022; Ramos et al. 2026).

Genomic comparisons have identified avian isolates that are closely related to bacteria recovered from environmental, livestock, and companion-animal sources. They have also revealed shared resistance determinants and plasmid-associated regions among isolates from different sources. However, genomic similarity alone does not establish transmission or its direction. The strength of such inferences depends on genome-wide relatedness, sampling density, temporal and spatial concordance, and the availability of isolates from plausible source populations. Studies based on isolated sampling events or incomplete source populations have more limited capacity to distinguish recent transmission from wider circulation of established bacterial lineages.

## Antimicrobial importance and One Health implications

From an ornithological perspective, AMR in birds reflects the ecological and behavioral diversity of avian orders. Synanthropic species may serve as sentinels of human-impacted contamination, while aquatic and migratory birds may provide insights into the geographic connectivity of resistant microorganisms. Predatory and scavenging species may encounter antimicrobial-resistant bacteria through trophic and environmental exposure pathways. Viewing AMR through this ecological and One Health approach highlights the value of birds as sentinels of environmental contamination and as hosts in which antimicrobial-resistant bacteria can be detected. However, the ecological significance of these findings depends on the characteristics of the sampled populations, the methods used to detect resistance, and the availability of epidemiological or genomic information linking bacterial populations across hosts and environments. Accordingly, evidence of bacterial carriage should be distinguished from evidence of acquisition from a specific source, persistence or maintenance within avian populations, geographic dissemination, and direct transmission between birds and other hosts or environments. Across ecological contexts, antimicrobial-resistant Gram-negative bacteria have been reported in urban and synanthropic, aquatic and migratory, agricultural, captive and rehabilitation, and relatively remote avian populations. However, the available evidence does not support a consistent ranking of these settings according to AMR burden, nor does it demonstrate a systematic difference between wild and captive birds, because studies differ substantially in host species, sampling design, bacterial targets, antimicrobial panels, and molecular methods. In captive and rehabilitation settings, antimicrobial exposure and management conditions may contribute to observed resistance patterns, whereas in free-living birds, environmental and anthropogenic exposure may be more relevant. Similarly, although antimicrobial-use practices may influence resistance within particular agricultural or managed settings, the available studies do not provide sufficiently comparable country- or region-level antimicrobial-use data to support direct conclusions regarding regional spillover into wild bird populations. Therefore, investigating antimicrobial-resistant bacteria in wild and captive birds remains important for advancing our understanding of resistance ecology and its implications for human, animal, and ecosystem health within the One Health framework. Future studies should prioritize standardized antimicrobial susceptibility testing, longitudinal sampling over time, and sampling of birds together with relevant environmental and animal-associated compartments, combined with genomic characterization, to better distinguish carriage from persistence, geographic connectivity, and transmission.

## Limitations of the present synthesis

The evidence synthesized in this review is heterogeneous in terms of study design, avian taxa, geographic representation, sampling strategies, antimicrobial panels, interpretive breakpoints, MDR/XDR definitions, and antimicrobial susceptibility-testing methods. The predominance of cross-sectional studies, inconsistent reporting of denominators, reliance on culture-dependent detection strategies, and uneven availability of genomic data further limit direct comparisons across avian populations and ecological settings.

In addition, because this is a narrative review, no formal systematic-review screening process, risk-of-bias assessment, or quantitative meta-analysis was performed. The literature-identification process may therefore be subject to publication, selection, and language-related biases. Accordingly, the conclusions should be interpreted as an evidence-informed conceptual synthesis rather than as pooled estimates of AMR prevalence or transmission risk.

## Supplementary Information

Below is the link to the electronic supplementary material.

Supplementary file1 (XLSX 50 KB)

## Data Availability

No datasets were generated or analysed during the current study.
